# Correlation Between Geometric Parameters and Capacitance in Silicon Detectors: A Study Based on Physical Modeling, Simulation, and Experiment

**DOI:** 10.3390/mi17080888

**Published:** 2026-07-25

**Authors:** Xinqing Li, Tao Long, Jun Zhao, Shunmao Lu, Yongguang Xiao, Zheng Li

**Affiliations:** 1 School of Materials Science and Engineering, Xiangtan University, Xiangtan 411105, China; xqli1996zz@163.com (X.L.); ygxiao@xtu.edu.cn (Y.X.); 2 Shandong Provincial Engineering Research Center for Optoelectronic Sensing Materials and Device Micro-Nano Manufacturing, School of Integrated Circuits, Ludong University, Yantai 264025, China; longtao@ldu.edu.cn (T.L.); zhaojun@ldu.edu.cn (J.Z.); lsm370686003x@163.com (S.L.); 3 Engineering Research Center of Photodetector Special Chip in Universities of Shandong, Ludong University, Yantai 264025, China

**Keywords:** silicon detectors, geometry-based capacitance model, SDD anode capacitance, TCAD simulation

## Abstract

This study proposes and validates a unified geometry-based capacitance model for four representative silicon detector architectures: planar, 3D trench electrode, 3D spherical electrode, and silicon drift detector (SDD). Closed-form analytical expressions explicitly relate capacitance to key geometric parameters—anode radius, depletion thickness, electrode depth, and electrode spacing—and the resulting geometric scaling laws are rigorously verified by combining physical modeling, TCAD simulation, and experimental measurement. A central finding is that for highly symmetric structures, capacitance is governed almost exclusively by the radius of the collecting anode and is essentially independent of the overall detector volume, thereby defining an ideal low-capacitance limit. For the SDD, a hemispherical capacitor approximation accurately captures this anode-dominated behavior, and measurements on prototypes together with independent literature data confirm that the total capacitance can be decomposed into an intrinsic geometric component and a parasitic contribution. This work provides a unified framework and direct cross-structure design guidelines for minimizing capacitance toward ultra-low-noise, high-performance silicon detectors.

## 1. Introduction

Silicon detectors have become fundamental components in various disciplines, including high-energy physics [1], space exploration [2], and radiation imaging [3]. Low-capacitance design serves as the foundational physical principle for achieving high-performance detection, as it directly influences core metrics such as energy resolution and count rate [4]. Since the equivalent noise charge (ENC) of a detector is proportional to the square of its total input capacitance [5], minimizing capacitance is mandatory for high-resolution and high-rate operation. This requirement has been further intensified by emerging applications—such as dark matter direct detection [6], quantum computing readout [7], and high-resolution photon-counting computed tomography (CT) [8]—where sub-femtofarad (sub-fF) anode capacitance has become a critical performance bottleneck.

The capacitance of a silicon detector is not a constant; it is determined by the detector’s physical structure, material properties, and operating conditions [9]. Among these factors, geometry—including electrode dimensions, depletion-layer thickness, and electrode layout—is the most fundamental [10]. Although many studies have documented the capacitance characteristics of silicon detectors, most rely on TCAD simulations or limited experimental measurements and lack a systematic capacitance model based on geometric parameters [11,12]. In recent years, several research directions have further highlighted the need for such a model. Machine learning and artificial intelligence techniques have been increasingly adopted to rapidly predict capacitance for complex, non-standard geometries, thereby shortening optimization cycles [13,14].

The growing trend toward monolithic integration of readout electronics (ASICs) with detector substrates has drawn attention to parasitic capacitances arising from interconnects, guard rings, and bonding pads, which can dominate the total capacitance in state-of-the-art systems [15,16]. Moreover, novel fabrication techniques—such as deep reactive ion etching (DRIE) and advanced epitaxial growth—have enabled practical implementations of previously theoretical geometries (e.g., quasi-spherical and nested 3D trench electrodes), raising new challenges for capacitance modeling [17,18]. Despite these advances, a unified analytical framework that directly links capacitance to geometric parameters across fundamentally different detector architectures is still missing. Consequently, the distinct geometric scaling laws that govern planar, 3D trench, 3D spherical, and silicon drift detectors (SDDs) have not been examined under a common theoretical perspective.

In this work, we develop a unified geometry-based capacitance model that simultaneously applies to these four representative silicon detector structures. By applying Gauss’s law to simplified yet physically representative electrostatic configurations, we derive explicit analytical expressions that relate capacitance to key geometric variables: anode radius, detector thickness, electrode depth, and electrode spacing. A central finding is that for highly symmetric configurations—such as 3D spherical electrode detectors and SDDs—the capacitance becomes dominated by the minimal anode radius and is essentially independent of the overall detector volume.

For the SDD, we further demonstrate that a hemispherical capacitor approximation accurately captures this anode-limited behavior, offering both clear physical insight and a simple predictive formula. The derived scaling laws are systematically verified through Synopsys Sentaurus TCAD simulations covering wide parameter ranges for all four structures. To bridge theory and practice, capacitance measurements are performed on two fabricated SDD prototypes. The experimental results confirm the dominant role of the anode radius and allow the total capacitance to be decomposed into an intrinsic geometric component and a nearly constant parasitic offset. By integrating physical modeling, TCAD simulation, and experimental validation, this study provides a direct cross-structure comparison and establishes practical design guidelines for minimizing capacitance toward ultra-low-noise silicon detector operation.

## 2. Physical Modeling and Analytical Calculation of Capacitance

### 2.1. Planar Detectors: Parallel-Plate Capacitor Model

**Figure 1:**
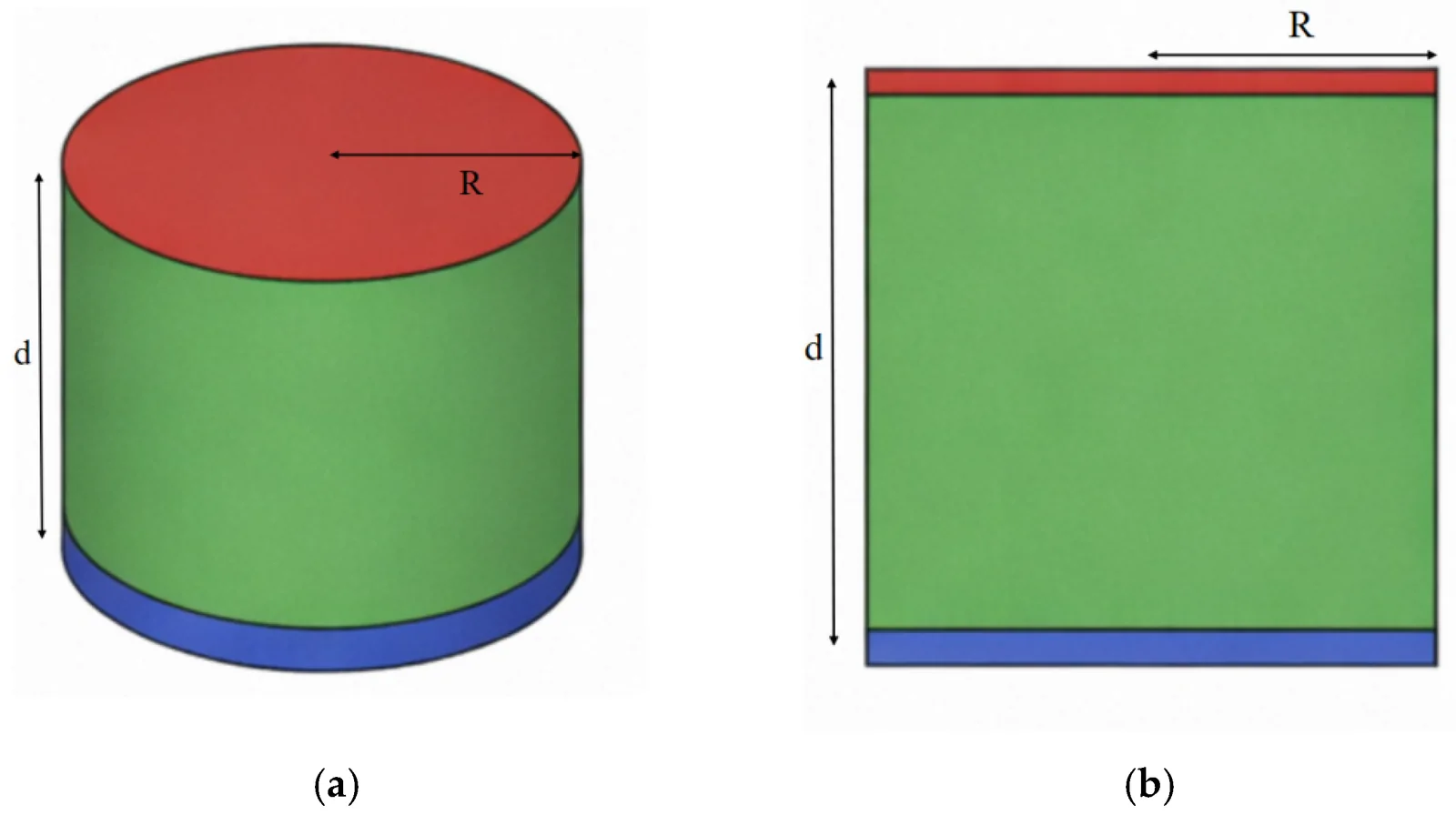
Schematic of a circular parallel-plate detector: (**a**) 3D view and (**b**) cross-sectional view. The anode radius R, electrode spacing d are indicated.

As a fundamental illustrative model for understanding the working principle of silicon detectors, we consider the parallel-plate detector [19]. As shown in Figure 1a, a circular parallel-plate capacitor is characterized by three geometric parameters: the electrode radius R, the inter-electrode distance d, and the permittivity ε of the semiconductor material filling the space between the electrodes. Under the assumptions that the detector is fully depleted and that fringing fields at the electrode edges can be neglected, the detector capacitance can be calculated analytically. In this configuration, the area A of the circular collecting anode is $A\;=\;\pi R^{2}$, as illustrated in Figure 1b.

Subsequently, in accordance with Gauss’s law:

(1) $$E\left(r\right)\cdot 2\pi R^{2}=\frac{2Q_{a}}{{\varepsilon \varepsilon }_{0}}$$

where $Q_{a}$ is the terminal charge on the collecting anode. In a fully depleted detector, the depletion region contains fixed ionized dopants with a net charge $Q_{dep}$ and a volume charge density $\rho =Q_{dep}∕\left(\pi R^{2}d\right)$. By charge neutrality and Gauss’s law, the anode charge and the depletion charge are equal in magnitude and opposite in sign, i.e., $Q_{a}=\left|Q_{dep}\right|$. Crucially, the capacitance is fundamentally defined in terms of the terminal charge $Q_{a}$, not the fixed charge $Q_{dep}$, because only $Q_{a}$ responds to the small-signal voltage variation.

The electric field strength between the plates is therefore given by:

(2) $$E\left(r\right)=\frac{Q_{a}}{{\varepsilon \varepsilon }_{0}\cdot \pi R^{2}}=\frac{V}{d}$$

Thus, the detector’s geometric capacitance C is defined as the ratio of terminal charge $Q_{a}$ to the potential difference V:

(3) $$C=\frac{Q_{a}}{V}=\frac{{\varepsilon \varepsilon }_{0}}{d}\cdot \pi R^{2}$$

The analytical model of the parallel-plate capacitor demonstrates that the capacitance C of a fully depleted planar detector is inversely proportional to the depletion thickness d and proportional to the square of the anode radius R (i.e., to the anode area). This model not only provides an intuitive physical picture and a useful first-order approximation for analyzing the capacitance of 2D planar silicon detectors, but also establishes a theoretical foundation and a performance benchmark for understanding the capacitance characteristics of more complex planar detector structures, such as silicon strip detectors (SSDs) and silicon pixel detectors (SPDs). The explicit dependence of capacitance on electrode area and detector thickness revealed by this model offers a key design guideline for achieving low-capacitance, high-performance operation.

### 2.2. 3D Trench Electrode Detector: Cylindrical Capacitor Model

The capacitance of conventional 2D planar detectors is well described by the parallel-plate model. Increasing the depletion thickness d reduces the geometric capacitance, thereby lowering the series noise and improving the signal-to-noise ratio [20]; however, a thicker detector also increases the carrier drift time and the full-depletion voltage. Conversely, reducing the thickness enables fast charge collection and improves radiation hardness, but at the expense of higher capacitance. This inherent trade-off limits the simultaneous optimization of noise and speed in planar geometries.

**Figure 2:**
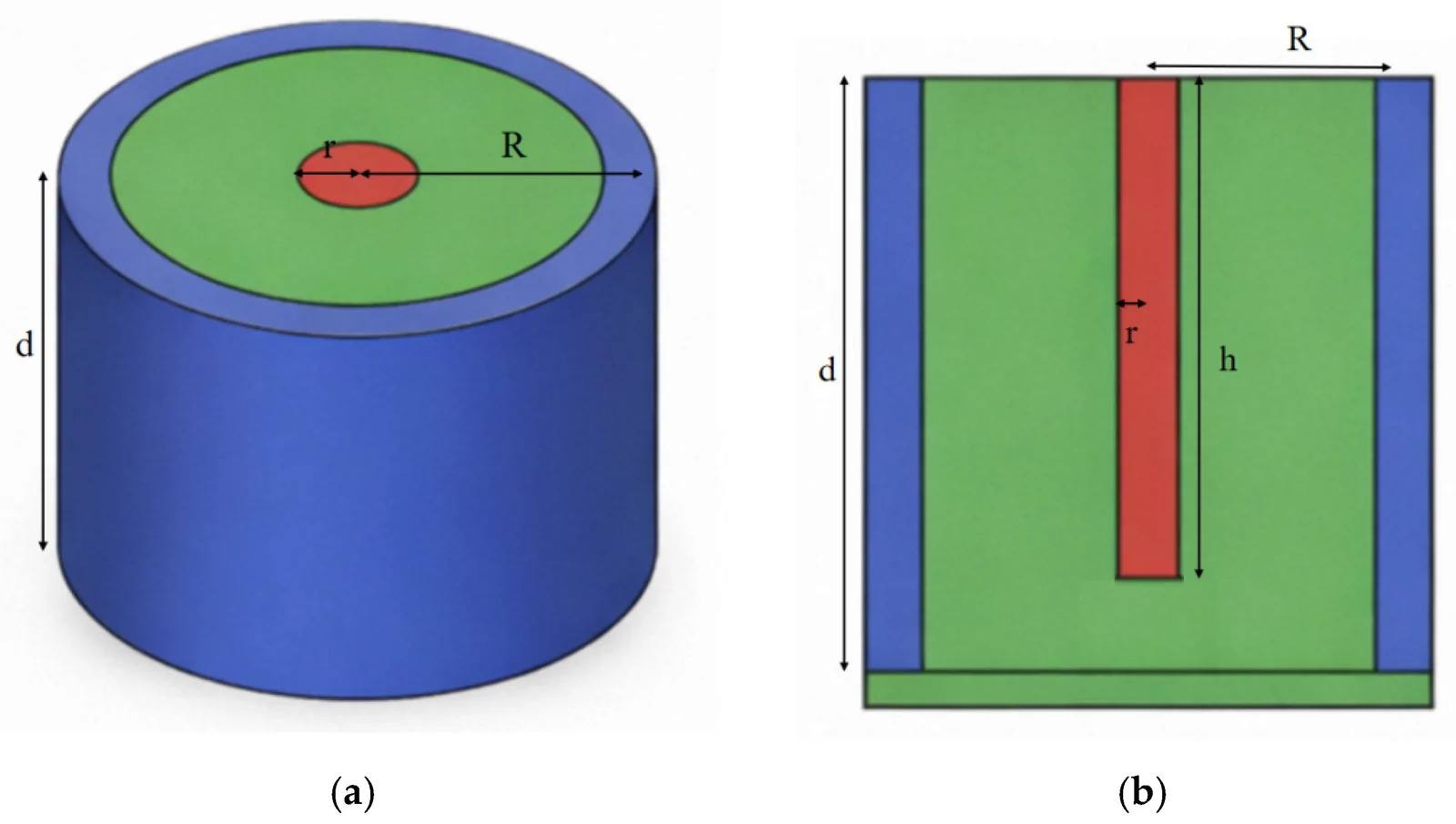
Schematic of a 3D trench electrode detector: (**a**) 3D view and (**b**) cross-sectional view.

In contrast, 3D trench electrode detectors decouple the electrode spacing from the substrate thickness by employing vertically oriented cylindrical, hexagonal, or square trench electrodes. As illustrated in Figure 2, their capacitance is governed by a coaxial-wire model [21], which differs fundamentally from the parallel-plate model because the electric field distribution is cylindrical rather than planar, owing to the vertical electrode configuration.

To illustrate this, consider a cylindrical electrode structure. According to Gauss’s theorem:

(4) $$E\left(r\right)\cdot 2\pi rh=\frac{Q_{a}}{{\varepsilon \varepsilon }_{0}}$$

where $Q_{a}$ is the terminal charge on the central anode. As in the planar case, under full depletion, $Q_{a}$ equals the magnitude of the total ionized dopant charge $Q_{dep}$ in the cylindrical depletion volume, but the capacitance is defined via $Q_{a}$.

Thus, we obtain:

(5) $$E\left(r\right)=\frac{Q_{a}}{{\varepsilon \varepsilon }_{0}}\cdot \frac{1}{2\pi rh}$$

The potential between the electrodes is then given by:

(6) $$V=\int_{r_{c}}^{R}E\left(r\right)dr=\frac{Q_{a}}{2\pi {\varepsilon \varepsilon }_{0}h}\cdot ln\frac{R}{r_{c}}$$

where R is the inner radius of the outer cathode electrode, the geometric capacitance of the detector can be derived as:

(7) $$C=\frac{Q_{a}}{V\left(r\right)}=\frac{{2\pi \varepsilon \varepsilon }_{0}h}{ln\frac{R}{r_{c}}}\left(0<h\leq d\right)$$

According to the cylindrical capacitor model, the capacitance of a 3D trench detector exhibits a linear dependence on the electrode column depth h. Because the effective electrode area increases with h, a deeper column directly raises the capacitance; conversely, if h is kept constant, the capacitance remains essentially unchanged even when the total substrate thickness is increased substantially. The same model further shows that C increases with the central anode radius $r_{c}$ and decreases with the electrode spacing g ($g=R-r_{c}$), but both dependencies are logarithmic and therefore comparatively weak. Consequently, although g reduces the capacitance, the effect is limited and saturates progressively. Crucially, this logarithmic scaling enables a favorable trade-off unique to 3D trench detectors: by designing the electrode spacing g to be very small (e.g., 50 μm), a strong electric field can be established for rapid charge collection, while the logarithmic dependence effectively suppresses any sharp rise in capacitance, allowing low capacitance and low full-depletion voltage to be achieved simultaneously.

### 2.3. 3D Spherical Electrode Detector: Spherical Capacitor Model

**Figure 3:**
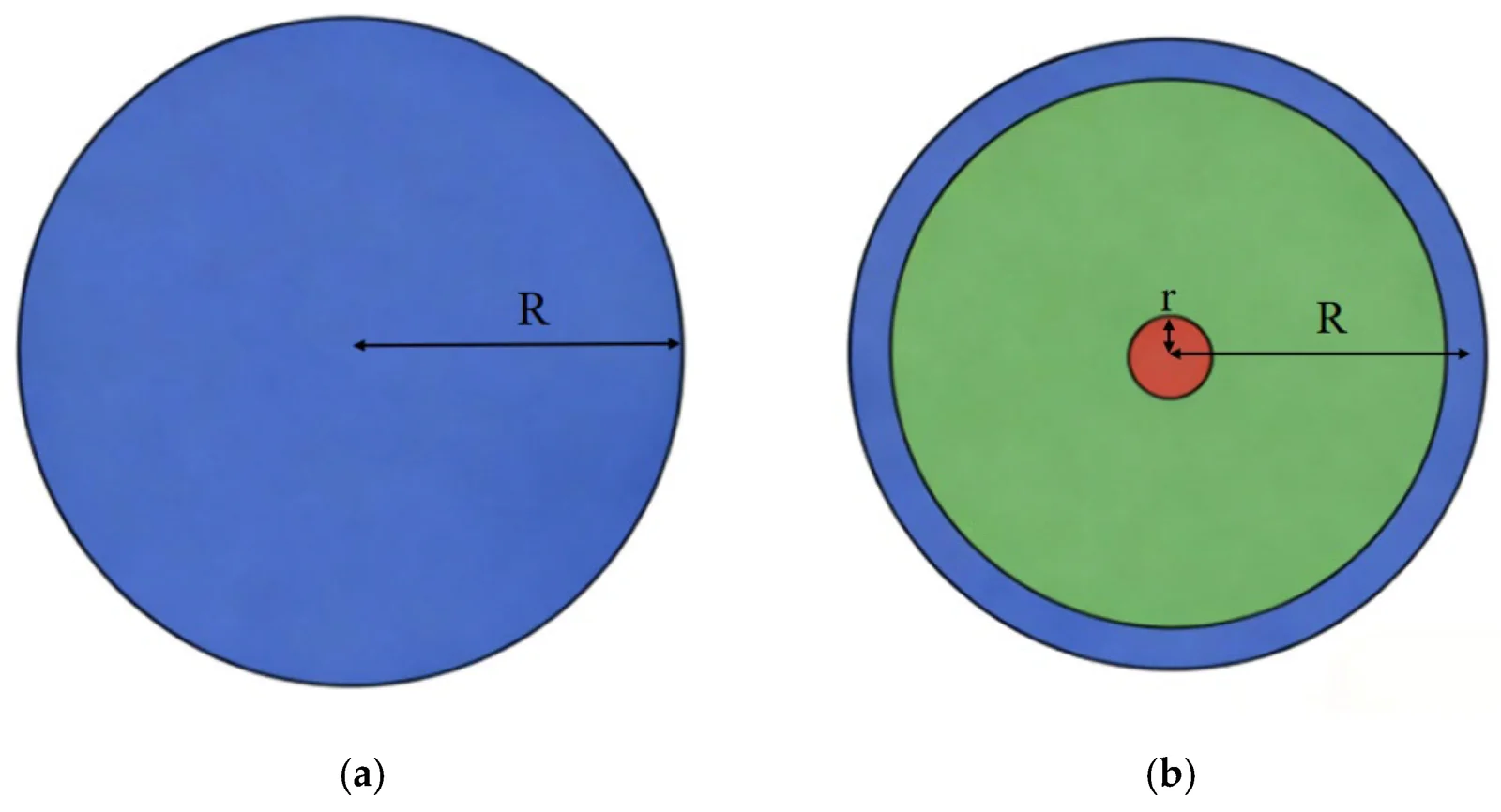
Schematic of a 3D spherical-electrode detector: (**a**) 3D view and (**b**) cross-sectional view.

The 3D spherical electrode silicon detector is characterized by an ideal spherical symmetry, which endows it with a set of unique and advantageous electrical properties. A schematic of the structure is shown in Figure 3. When a bias voltage is applied to the concentric spherical electrodes, a purely radial electric field is established within the fully depleted silicon bulk. All field lines are directed radially outward (or inward), and the field strength depends exclusively on the radial distance from the center. As a result, regardless of the position at which an incident particle generates electron–hole pairs, the charge carriers drift along straight radial paths toward the corresponding electrodes, experiencing no lateral deflection.

The 3D spherical electrode detector features a radial electric field $E_{r}\propto 1/r^{2}$, which directs carriers along straight radial paths toward the electrodes with no lateral deflection. The field strength peaks sharply near the small collecting anode, ensuring rapid carrier acceleration in the final stage of collection. Although the field varies more rapidly with radius than in planar or cylindrical geometries, the combination of short collection distance and high near-anode field results in efficient overall charge collection—an advantage for radiation-hard applications where fast transit near the anode reduces trapping risk. Despite fabrication challenges, quasi-spherical structures [22,23] offer practical alternatives that preserve these key electrostatic benefits.

To establish the analytical capacitance model, we consider an ideal spherical electrode geometry: a small doped anode (e.g., n^+^) of radius $r_{c}$ is embedded in a uniform silicon medium and concentrically surrounded by a doped shell (e.g., p^+^) with an outer radius R, as illustrated in Figure 3b.

The potential difference between the electrodes is given by:

(8) $$V=-\int_{r_{c}}^{R}E\left(r\right)dr$$

According to Gauss’s theorem:

(9) $$E\left(r\right)\cdot 4\pi r^{2}=\frac{Q_{a}}{{\varepsilon \varepsilon }_{0}}$$

where $Q_{a}$ is the terminal charge on the spherical anode. Under full depletion,$\;\left|Q_{a}\right|=\left|Q_{dep}\right|$, where $Q_{dep}$ is the total ionized dopant charge within the spherical depletion shell.

The obtained potential is:

(10) $$V=\frac{Q_{a}}{{4\pi \varepsilon \varepsilon }_{0}}\cdot \left(\frac{1}{r_{c}}-\frac{1}{R}\right)$$

From this, the capacitance of the 3D spherical electrode detector is derived as:

(11) $$C=\frac{Q_{a}}{V}=\frac{{4\pi \varepsilon \varepsilon }_{0}}{\left(\frac{1}{r_{c}}-\frac{1}{R}\right)}≅{4\pi \varepsilon \varepsilon }_{0}r_{c}\;\;\;\left(R\gg r_{c}\right)$$

Unlike planar detectors, the capacitance of an ideal spherical electrode depends solely on the radius of its central collection anode and the permittivity of the silicon medium. This direct and explicit relationship provides a clear physical basis for precisely designing detectors to achieve target capacitance values. Although idealized, this model establishes a theoretical foundation for understanding the capacitance of more complex 3D spherical electrode configurations and offers valuable guidance for modern detector designs requiring low capacitance and fast response.

### 2.4. Silicon Drift Detector: Method of Images and Hemispherical Approximation

Silicon drift detectors (SDDs) achieve excellent energy resolution at room temperature owing to their unique lateral drift-field design, and are consequently widely used in material analysis, space exploration, and medical imaging [24]. This performance stems primarily from their very low anode capacitance, which minimizes electronic noise and enables both high energy resolution (122–125 eV for Fe^55^ X-rays) and high counting rate capability [25]. These dual characteristics are essential to meet the high precision and high throughput requirements of modern analytical instrumentation.

**Figure 4:**
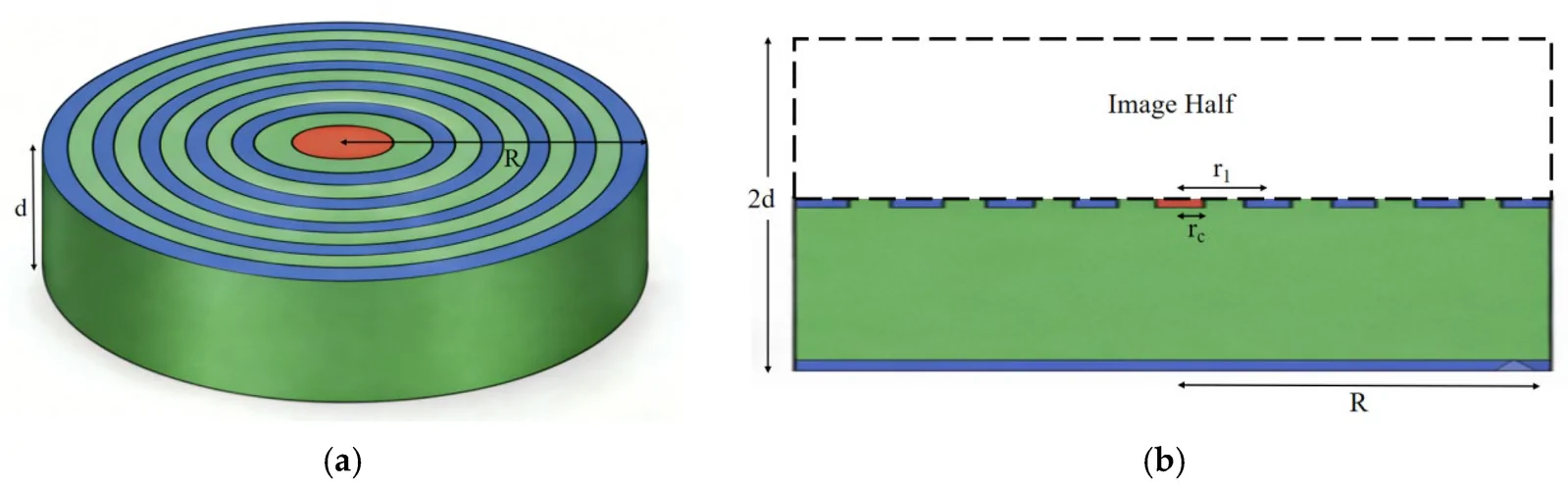
Schematic of a Silicon drift detector: (**a**) 3D view and (**b**) cross-sectional view.

As shown in Figure 4, the SDD features a small central anode of radius $r_{c}$, while the overall detector radius R is much larger (R ≥ $r_{c}$). The capacitive behavior can therefore be approximated by the 3D spherical electrode model. By applying the method of images—placing a mirrored SDD above the original, as illustrated in Figure 4b—the pair approximates a full sphere. Consequently, the SDD capacitance can be taken as half of the corresponding spherical capacitance.

Then, according to Gauss’s theorem (applied to the combined sphere formed by the mirror method):

(12) $$E\left(r\right)\cdot \left(2\pi r^{2}+2\pi r\cdot 2d\right)=\frac{2Q_{a}}{{\varepsilon \varepsilon }_{0}}$$

In the mirror-method picture, ${2Q}_{a}$ is the total terminal charge on the two anodes (the real SDD anode and its mirror), so $Q_{a}$ represents the terminal charge on the SDD anode itself. Under full depletion, $\left|Q_{a}\right|=\left|Q_{dep}\right|$ in the corresponding drift region.

Solving for the electric field strength, we obtain:

(13) $$E\left(r\right)=\frac{Q_{a}}{\pi {\varepsilon \varepsilon }_{0}\left(r^{2}+2rd\right)}$$

The potential between the electrodes is then given by:

(14) $$V=\int_{r_{c}}^{R}E\left(r\right)dr=\frac{Q_{a}}{\pi {\varepsilon \varepsilon }_{0}}\cdot \int_{r_{c}}^{R}\frac{dr}{r^{2}+2rd}$$

Therefore, the capacitance of the complete sphere is:

(15) $$2C_{SDD}=\frac{2Q_{a}}{V}=2\pi {\varepsilon \varepsilon }_{0}\cdot {\left(\int_{r_{c}}^{R}\frac{dr}{r^{2}+2rd}\right)}^{-1}$$

Rearranging gives the SDD capacitance:

(16) $$C_{SDD}=\pi {\varepsilon \varepsilon }_{0}\cdot {\left[\int_{r_{c}}^{R}\frac{dr}{r\left(r+2d\right)}\right]}^{-1}$$

Carrying out the integration, the exact solution is obtained:

(17) $$C_{SDD}=\frac{2\pi {\varepsilon \varepsilon }_{0}d}{ln\left[\frac{R\left(r_{c}+2d\right)}{r_{c}\left(R+2d\right)}\right]}$$

In all steps, $Q_{a}$ denotes the terminal charge on the SDD anode, and the capacitance is defined as ${C_{SDD}=Q}_{a}∕V$.

A simpler and more direct approximation treats the SDD central anode as a hemisphere of radius $r_{c}$ facing an infinitely large grounded plane, which represents the detector surface. This configuration is electrostatically rigorous when the anode is much smaller than both the detector thickness d and the outer radius (d, R ≥ $r_{c}$), so that the electric field near the anode is approximately radial and the detector surface behaves as an ideal ground plane. It is a standard result in electrostatics that the capacitance of a hemispherical conductor opposite an infinite conducting plane is exactly half that of a full sphere. Therefore, the capacitance of the SDD central anode can be approximated as (as given by Equation (11)):

(18) $$C_{SDD}≅{2\pi \varepsilon \varepsilon }_{0}r_{c}$$

As a result, under practical design conditions (d, R ≥ $r_{c}$), the capacitance of the SDD, like that of an ideal 3D spherical electrode detector, is determined predominantly by its anode radius. This anode-limited behavior provides the physical basis for the low-capacitance performance of SDDs.

It should be noted that the method-of-image construction assumes an infinite, perfectly conducting ground plane and a point-like anode, which oversimplifies the real SDD geometry. The actual boundary conditions involve a finite-sized detector surface with graded potentials on the drift rings, so the mirror-based derivation should be regarded as a first-order approximation. The hemispherical model (Equation (18)) provides a more accurate and physically transparent description, as confirmed by the TCAD simulations in Section 3.4.

## 3. TCAD Simulation and Data Analysis

In this section, the capacitance characteristics of the four representative detector structures—planar, trench electrode, spherical electrode, and silicon drift detector (SDD)—are systematically simulated using TCAD to validate the analytical models established in Section 2. Particular attention is given to the SDD, for which the hemispherical approximation and the method of images are critically examined.

The simulations are performed with Synopsys Sentaurus [26], a widely adopted semiconductor process and device simulation platform that provides a comprehensive workflow covering process simulation, device physics, electrical characterization, and reliability analysis. The Sentaurus Structure Editor (SDE) serves as the primary tool for constructing 3D device geometries, ranging from single devices to complex detector arrays. The meshed structures are then transferred to Sentaurus Device for numerical simulation of the electrical characteristics.

For all detectors, the bulk material was n-type high-resistivity silicon with a uniform doping concentration of $N_{d}=1\times 10^{12}\;{\mathrm{cm}}^{-3}$ (resistivity ≈ 10 kΩ·cm). The heavily doped n^+^ and p^+^ electrodes were implemented as constant doping profiles with an active concentration of $1\times 10^{18}\;{\mathrm{cm}}^{-3}$. Ohmic contacts were defined at all electrodes. The quasi-stationary DC bias was ramped from 0 V to a value exceeding the full-depletion voltage (typically −70 V applied to the cathode for the planar detectors, with analogous ranges for the other geometries). The full set of physical models activated in Sentaurus Device includes: Fermi–Dirac statistics, incomplete ionization, the OldSlotboom effective intrinsic density model, mobility models accounting for doping dependence, carrier–carrier scattering, high-field saturation (driven by quasi-Fermi gradients), and Enormal (Lombardi) surface degradation; and recombination models including doping-dependent Shockley–Read–Hall, Auger, trap-assisted Auger, and surface SRH recombination.

The capacitance was extracted using the small-signal AC analysis module of Sentaurus Device, where a small AC voltage signal (amplitude 10 mV, frequency 1 MHz) was superimposed on the DC bias, and the terminal capacitance was derived from the imaginary part of the admittance, C = Im(Y)/ω.

The mesh was refined to a minimum element size of 0.1 µm in the vicinity of the p–n junctions and electrode edges, with a global maximum element size of 30 µm. A dedicated mesh-convergence study was performed by systematically reducing the local mesh size until the simulated full-depletion capacitance changed by less than 1%, confirming that the results are mesh-independent. The relative error between the analytical model $C_{\mathrm{model}}$ and the simulated value $C_{\mathrm{sim}}$, defined as $\delta \;=\;|C_{\mathrm{sim}}-C_{\mathrm{model}}|/C_{\mathrm{sim}}$, was below 5% for all detector types across the entire parameter range, with a typical average deviation of 2–3%. The specific geometric dimensions of each detector type are detailed in the respective subsections.

### 3.1. Planar Detector

As discussed in Section 2.1, the capacitance of a circular parallel-plate detector is primarily governed by the anode area (determined by the anode radius r) and the electrode spacing d. To systematically evaluate these dependencies, a silicon-based PIN circular parallel-plate structure was constructed using the Sentaurus SDE module, and parametric simulations were carried out with varying r and d.

**Figure 5:**
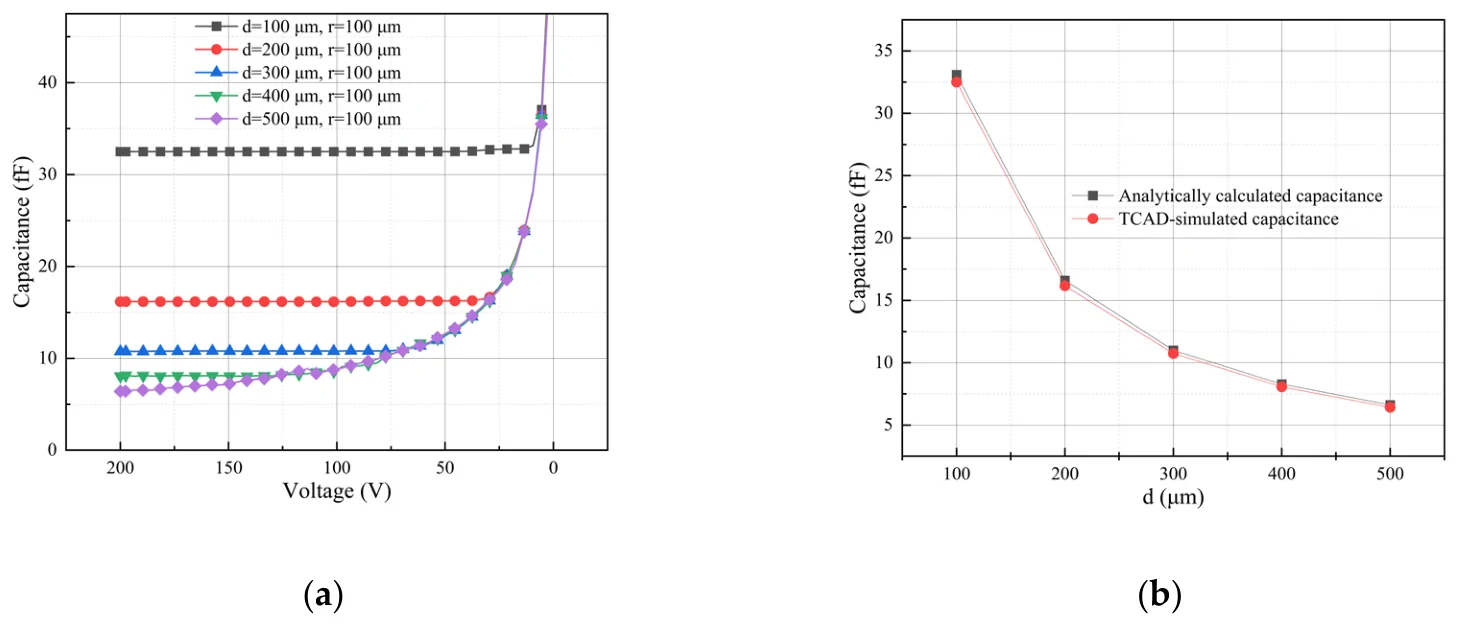
(**a**) C-V curves of the planar detector at different electrode spacings d. (**b**) Comparison between calculated and simulated capacitance values.

First, fixing r=100 μm, the electrode spacing was set to 100, 200, 300, 400, and 500 µm. The simulated C-V curves (Figure 5a) show that the capacitance decreases markedly with increasing d, in full agreement with the inverse proportionality predicted by the analytical model. The voltage at which the capacitance reaches its minimum marks the full-depletion voltage. For example, in Figure 5a with d=100 μm, this occurs at approximately −26 V. This voltage is given by $V_{\mathrm{FD}}=qN_{\mathrm{eff}}d^{2}/(2\varepsilon )$ and can be tuned by adjusting the substrate doping concentration or detector thickness, providing a direct means to balance operating voltage against detection efficiency. The quantitative agreement between simulation and theory is excellent (Figure 5b): as d increases fivefold from 100 µm to 500 µm, the capacitance drops from 33.1 fF to 6.62 fF, also by a factor of five.

**Figure 6:**
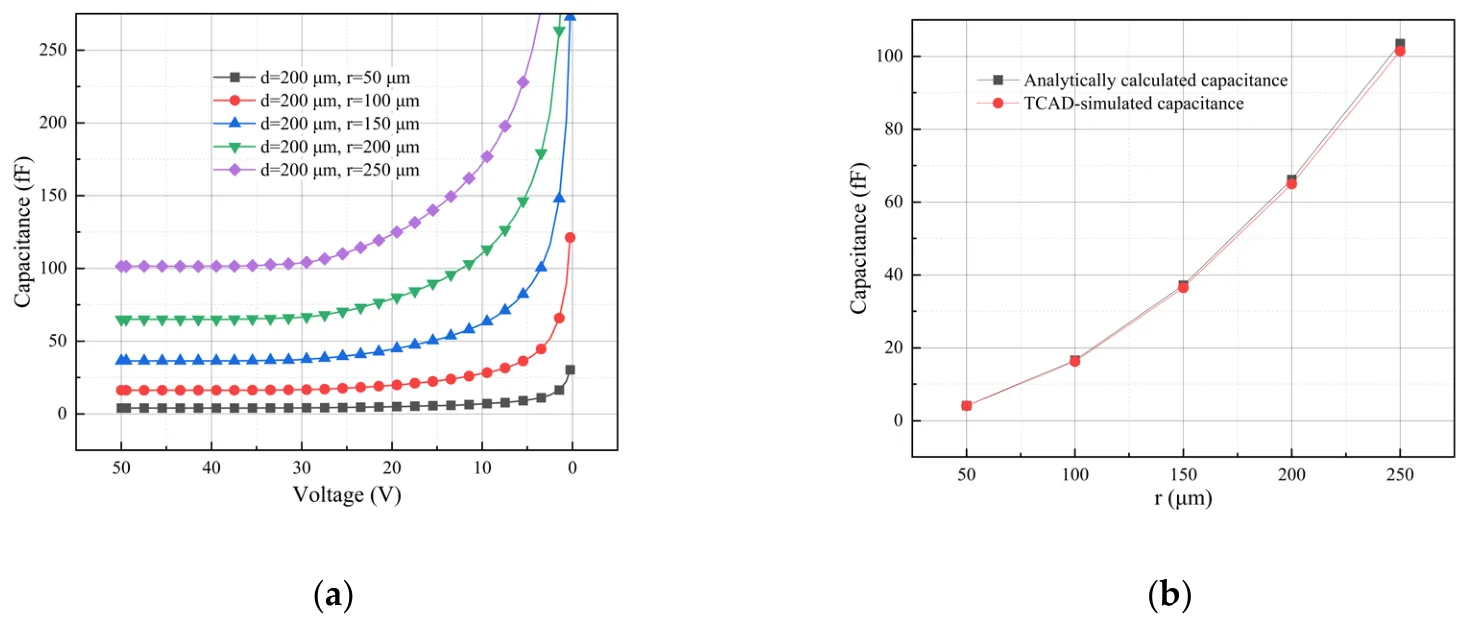
(**a**) C-V curves of the planar detector at different anode radius r. (**b**) Comparison between calculated and simulated capacitance values.

Second, for a fixed electrode spacing d=200 μm, the anode radius r was varied from 50 µm to 250 µm. The corresponding C-V curves (Figure 6a) reveal that the capacitance increases significantly with r, while the full-depletion voltage remains nearly unchanged. This demonstrates that reducing the anode area can lower the capacitance without raising the operating voltage—an important degree of freedom for low-noise design. The simulated values again match the theoretical predictions closely (Figure 6b). The data confirm that the capacitance scales with the square of the anode radius: as r increases fivefold from 50 µm to 250 µm, the capacitance rises from 4.14 fF to 103.5 fF, i.e., by a factor of approximately 25, consistent with $C\propto r^{2}$. This quadratic sensitivity implies that even a modest enlargement of the anode can lead to a substantial increase in capacitance, a fact that must be carefully considered in electrode design and process control, especially for high-precision detectors.

### 3.2. 3D Trench Electrode Detector

**Figure 7:**
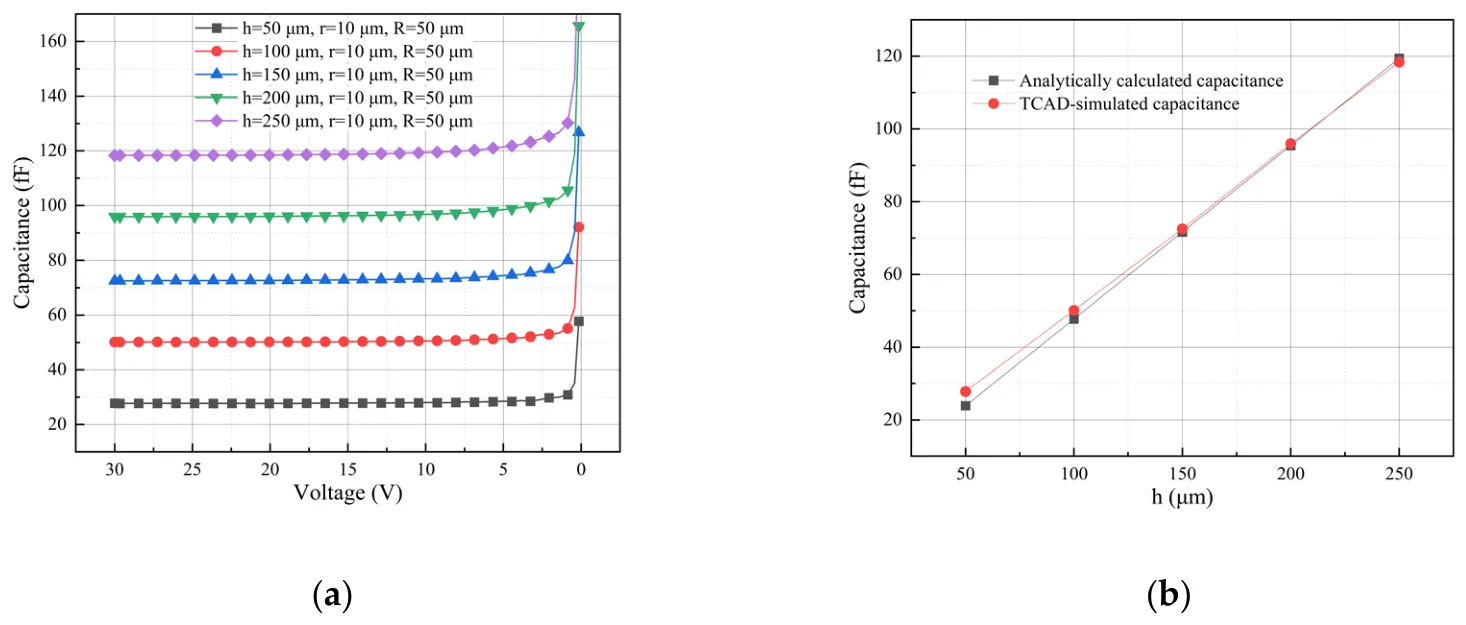
(**a**) C-V curves of the 3D trench electrode detector at different anode column depth h. (**b**) Comparison between calculated and simulated capacitance values.

**Figure 8:**
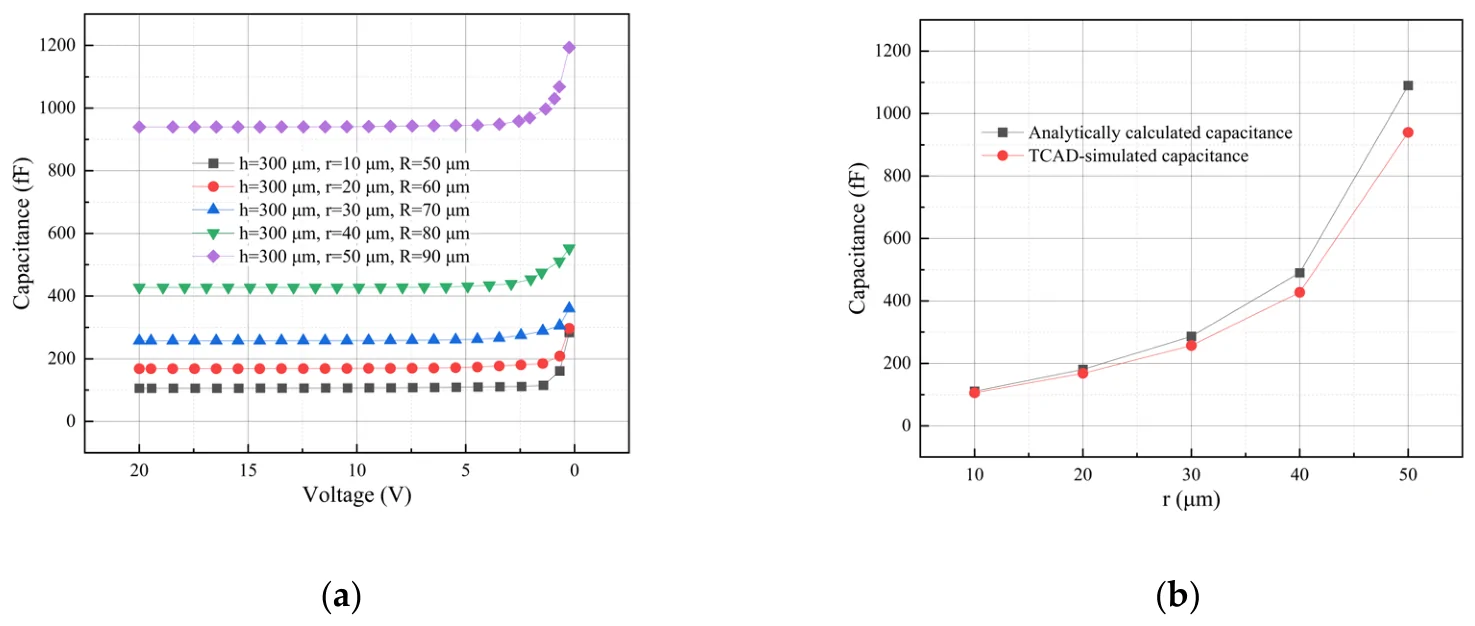
(**a**) C-V curves of the 3D trench electrode detector at different anode radius r. (**b**) Comparison between calculated and simulated capacitance values.

**Figure 9:**
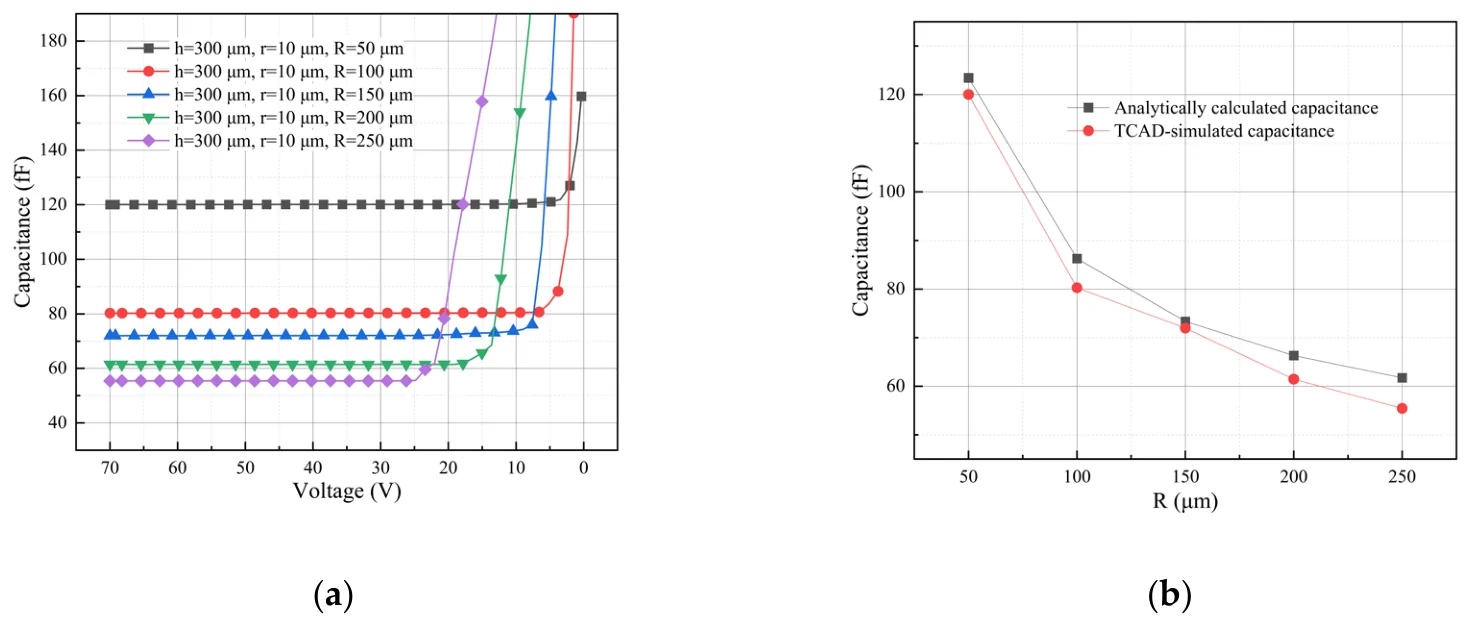
(**a**) C-V curves of the 3D trench electrode detector at different electrode spacing g. (**b**) Comparison between calculated and simulated capacitance values.

Based on the cylindrical capacitor model, the capacitance of a 3D trench electrode detector increases linearly with the electrode column depth h, increases with the central anode radius $r_{c}$, and decreases logarithmically with the electrode spacing g. Simulation results confirm these dependencies and show close quantitative agreement with the analytical expression across the entire parameter range, thereby validating the model (Figure 7, Figure 8 and Figure 9). Reducing h lowers the capacitance proportionally, while varying h or $r_{c}$ has negligible effect on the full-depletion voltage. In contrast, increasing g reduces the capacitance only weakly due to the logarithmic dependence $ln\;(1+g/r_{c})$, but it significantly raises the operating voltage required for full depletion.

The capacitance is most sensitive to the anode radius $r_{c}$: as $r_{c}$ increases from 10 μm to 50 μm, C rises from 102.1 fF to 590.5 fF, an increase by a factor of approximately 5.8. Hence, minimizing the anode radius is the most effective strategy for achieving low capacitance in 3D detectors. Together, these findings clearly illustrate the design trade-off between capacitance and operating voltage inherent to 3D trench structures, and provide key guidance for co-optimizing low capacitance and low full-depletion voltage.

### 3.3. 3D Spherical Electrode Detector

**Figure 10:**
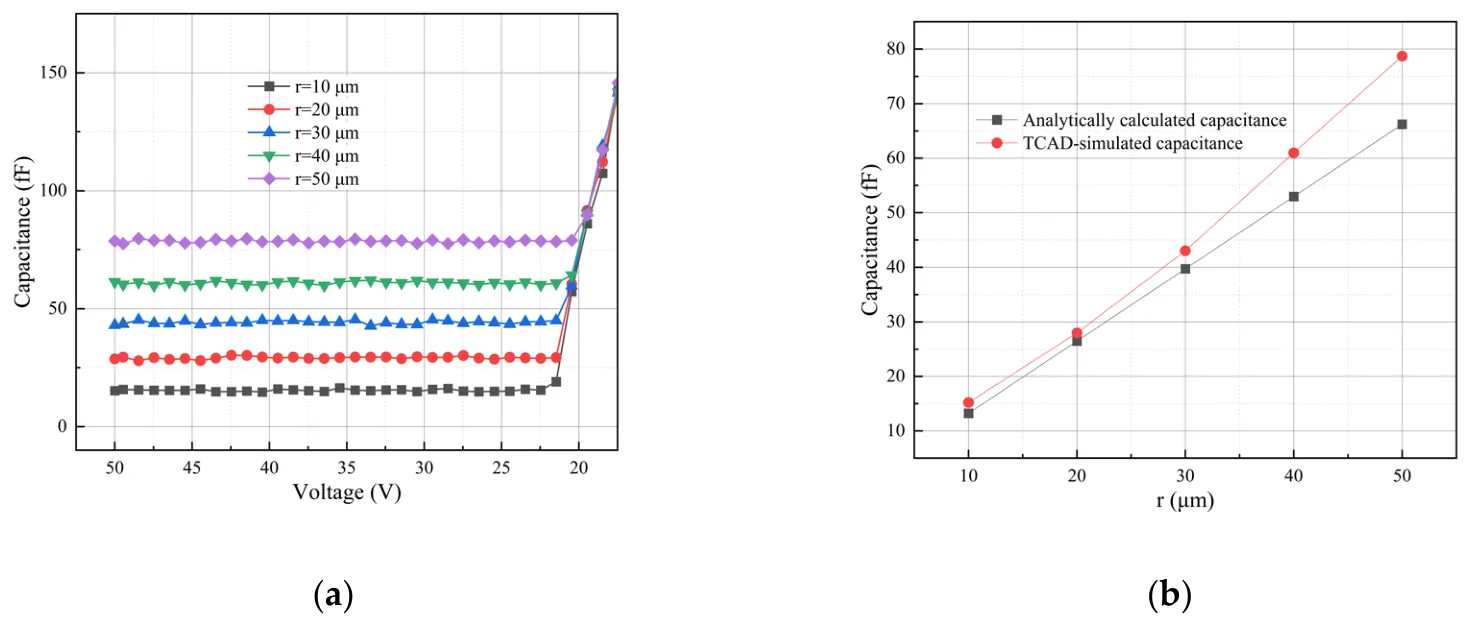
(**a**) C-V curves of the 3D Spherical Electrode Detectors at different anode radius r. (**b**) Comparison between calculated and simulated capacitance values.

For an ideal 3D spherical electrode detector, simulation results confirm that the capacitance scales linearly with the anode radius (Figure 10a). Specifically, increasing the radius from 10 μm to 50 μm (a factor of five) raises the capacitance from 13.24 fF to 66.20 fF—also by a factor of five—in full agreement with the theoretical prediction (Equation (11); Figure 10b). Although fabricating a perfectly spherical electrode remains challenging in practice, this idealized model defines the fundamental performance limit and provides essential theoretical guidance. The radial-field optimization principles derived from the spherical geometry can be transferred to practical designs: by tailoring the electrode layout to approximate a spherical configuration within feasible process constraints, a more favorable electric field distribution can be achieved.

In practical implementations, the spherical anode is commonly approximated by a planar pixel electrode (circular or square) on the detector surface, forming a quasi-hemispherical geometry. A circular pixel of radius $r_{p}$ generates an approximately hemispherical field, and its capacitance $C\approx 2\pi \varepsilon r_{p}$ is half that of an ideal full sphere of radius $r_{p}$. Hence, the pixel radius can be directly correlated with the equivalent spherical anode radius as $r_{\mathrm{pixel}}\approx r_{\mathrm{sphere}}$, providing a simple rule for mapping a target capacitance to a realizable electrode dimension.

### 3.4. Silicon Drift Detector

**Figure 11:**
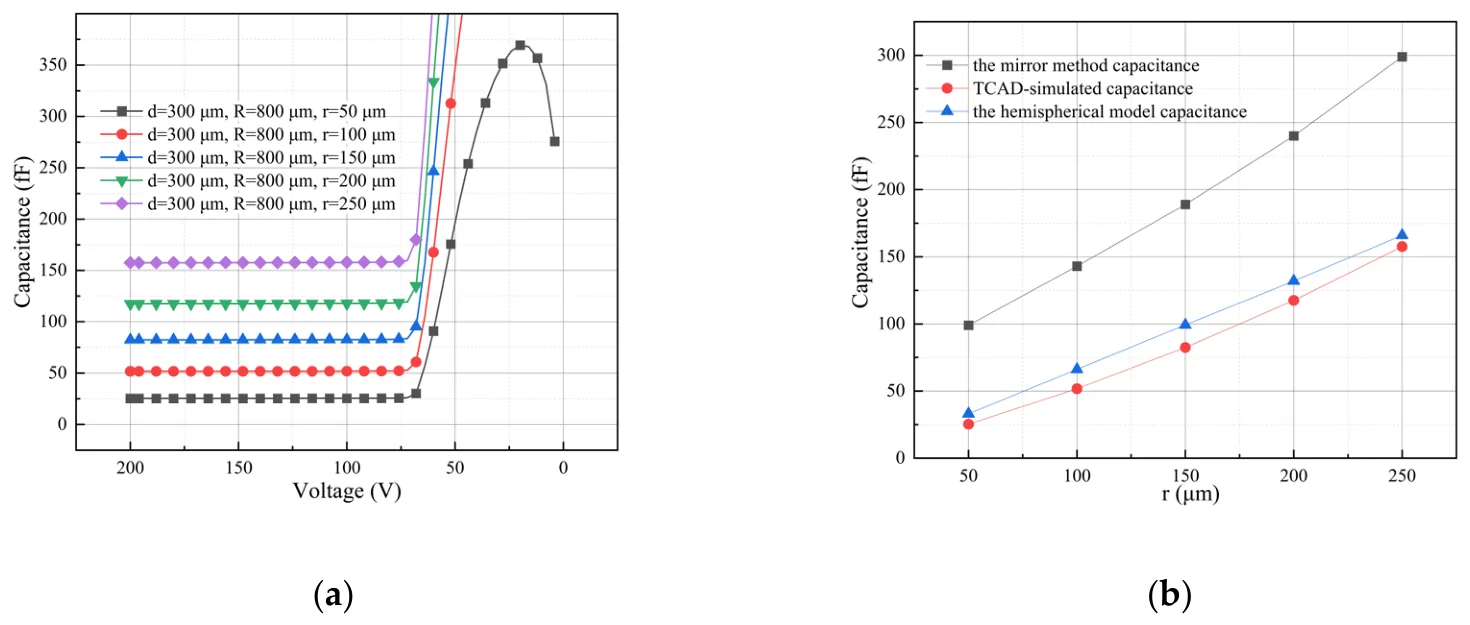
(**a**) C-V curves of the SDDs at different anode radius r. (**b**) Comparison between calculated and simulated capacitance values.

**Figure 12:**
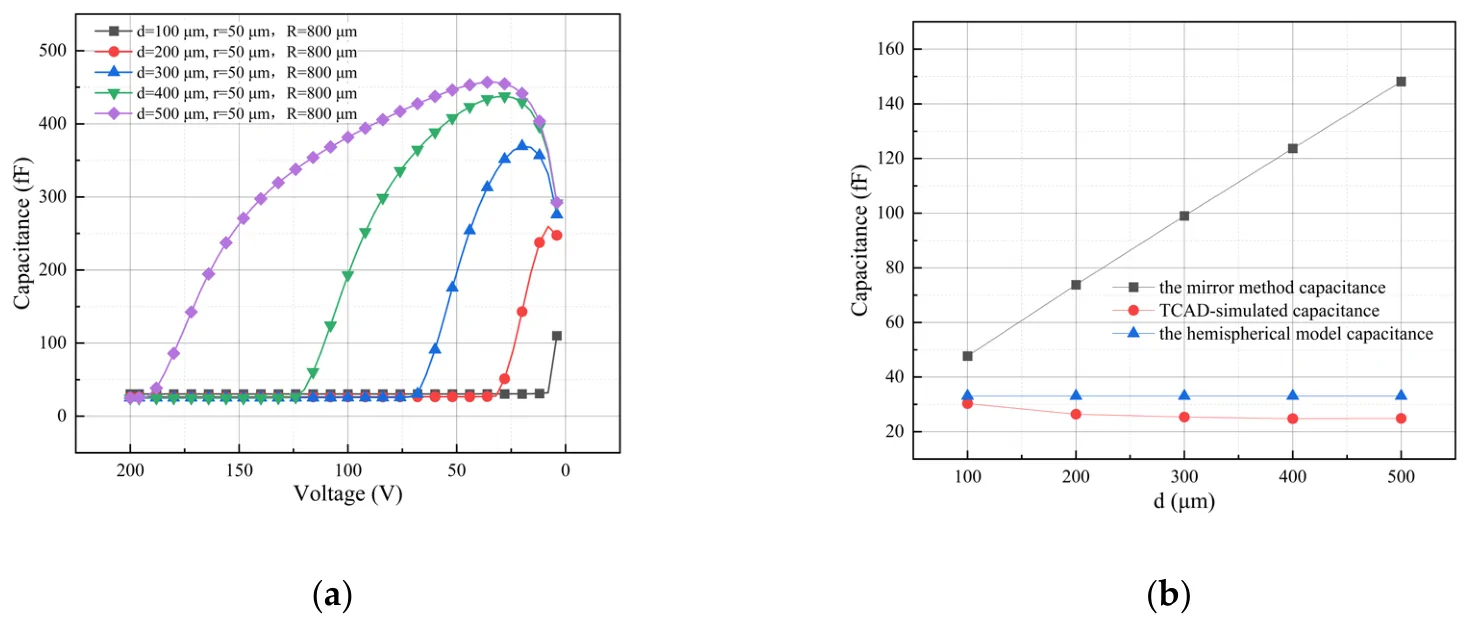
(**a**) C-V curves of the SDDs at different electrode spacing d. (**b**) Comparison between calculated and simulated capacitance values.

**Figure 13:**
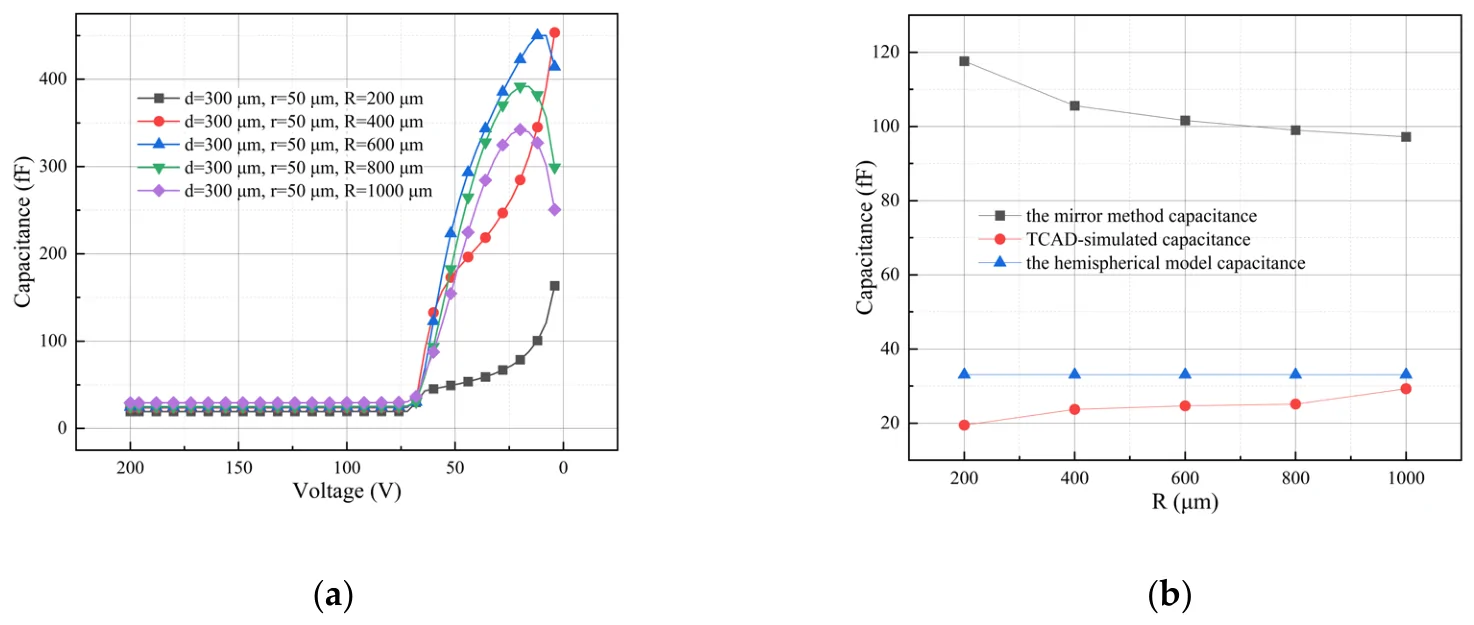
(**a**) C-V curves of the SDDs at different outer radius R. (**b**) Comparison between calculated and simulated capacitance values.

Simulations of the SDD confirm that its capacitance is governed primarily by the anode radius. As shown in Figure 11a, the capacitance scales directly with $r_{c}$: increasing $r_{c}$ from 50 μm to 250 μm raises the capacitance from 25.4 fF to 157.6 fF, i.e., by a factor of approximately six. As shown in Figure 12a and Figure 13a, the capacitance decreases gradually with bias and eventually stabilizes, indicating the transition from partial to full depletion. In an SDD, the full-depletion voltage is determined by the bulk doping concentration and the thickness of the drift region, analogous to the planar case. However, because the SDD employs a small central anode and distributed drift rings rather than a full-area electrode, the electric field distribution is inherently non-uniform, which tends to raise the full-depletion voltage compared with a planar detector of equivalent thickness. Larger electrode spacing or higher doping further increases the required operating voltage. This is particularly relevant for SDD design, as the drift rings must be appropriately biased to achieve full depletion across the entire active volume. Similarly, enlarging R from 200 μm to 1000 μm increases the capacitance by only about 10 fF, a negligible change (Figure 13a). These results indicate that, unlike planar or trench-electrode detectors, the SDD capacitance is essentially decoupled from the overall detector dimensions.

To further assess the analytical approximations, capacitance values obtained from the mirror method, the hemispherical capacitor model, and TCAD simulation were compared. Comparison of capacitance values obtained from three approaches: the mirror method (Equation (17)), the hemispherical capacitor approximation (Equation (18)), and TCAD simulation. The hemispherical model shows the closest agreement with simulation, confirming that it accurately captures the anode-dominated capacitance behavior of the SDD. Moreover, Figure 12b and Figure 13b demonstrate that the hemispherical model and the simulation both yield a capacitance that is virtually independent of d and R. In contrast, the mirror method predicts a capacitance that increases with d and decreases with R, deviating from the simulated behavior. This discrepancy arises because the mirror method effectively treats the SDD as a finite-sized parallel-plate system, which retains a residual dependence on the detector boundaries—an approximation that becomes inaccurate for a point-like or disk-shaped anode surrounded by large-area drift electrodes.

To quantify the validity range of the hemispherical approximation, the relative deviation $\delta =|C_{\mathrm{sim}}-C_{\mathrm{hemi}}|/C_{\mathrm{sim}}$ was evaluated as a function of the geometric ratios $r_{c}/R$ and $r_{c}/d$. For the simulated structures with $r_{c}/R<0.1$ and $r_{c}/d<0.1$, δ remains below 5%, confirming the practical accuracy of Equation (18) under typical SDD design conditions. As $r_{c}/R$ or $r_{c}/d$ increases beyond 0.1, the anode size becomes comparable to the detector boundaries, and the ideal ground-plane assumption is no longer strictly satisfied; consequently, the deviation grows. Nevertheless, the capacitance continues to scale nearly linearly with $r_{c}$ even outside the asymptotic regime, demonstrating that the anode radius remains the dominant geometric parameter.

Based on this analysis, the hemispherical capacitor model most accurately captures the quantitative relationship between structural parameters and capacitance. The SDD capacitance is determined predominantly by the anode radius, while other geometric factors are negligible. This insight provides a clear physical basis for design optimization and is further corroborated by the prototype measurements presented in the following section.

## 4. Experimental Validation and Discussion

### 4.1. SDD Prototype Parameters

Table 1 lists the parameters of SDDs tested in this work.

### 4.2. Measurement Setup

**Figure 14:**
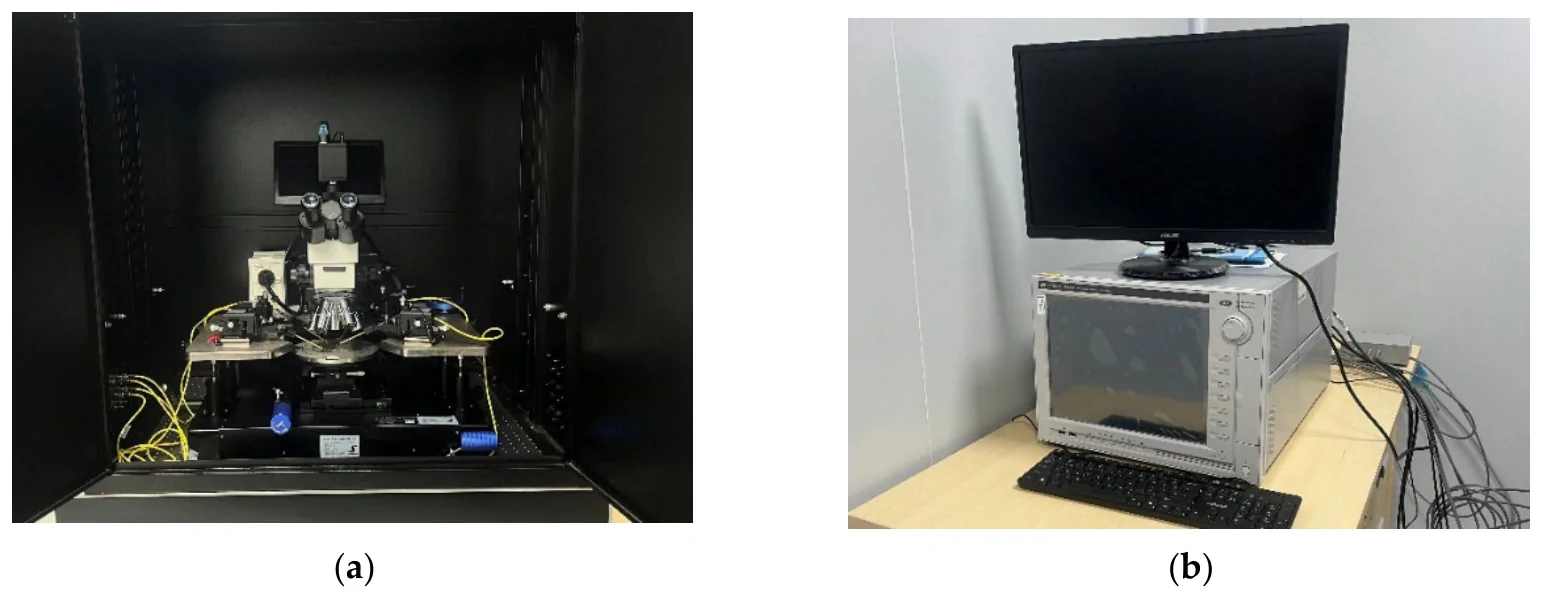
(**a**) CL-6 Probe Station in the dark/shielded chamber. (**b**) Semiconductor device analyzer.

Figure 14 shows the experimental setup at Ludong University for measuring SDD samples. The system integrates a CL-6 probe station, equipped with a CB-40-T holder and ST-20-0.5 probes, within a light-tight and electrically shielded enclosure, together with a semiconductor device analyzer, power supplies, and a high/low-temperature test chamber.

### 4.3. Discussion of Test Results

**Figure 15:**
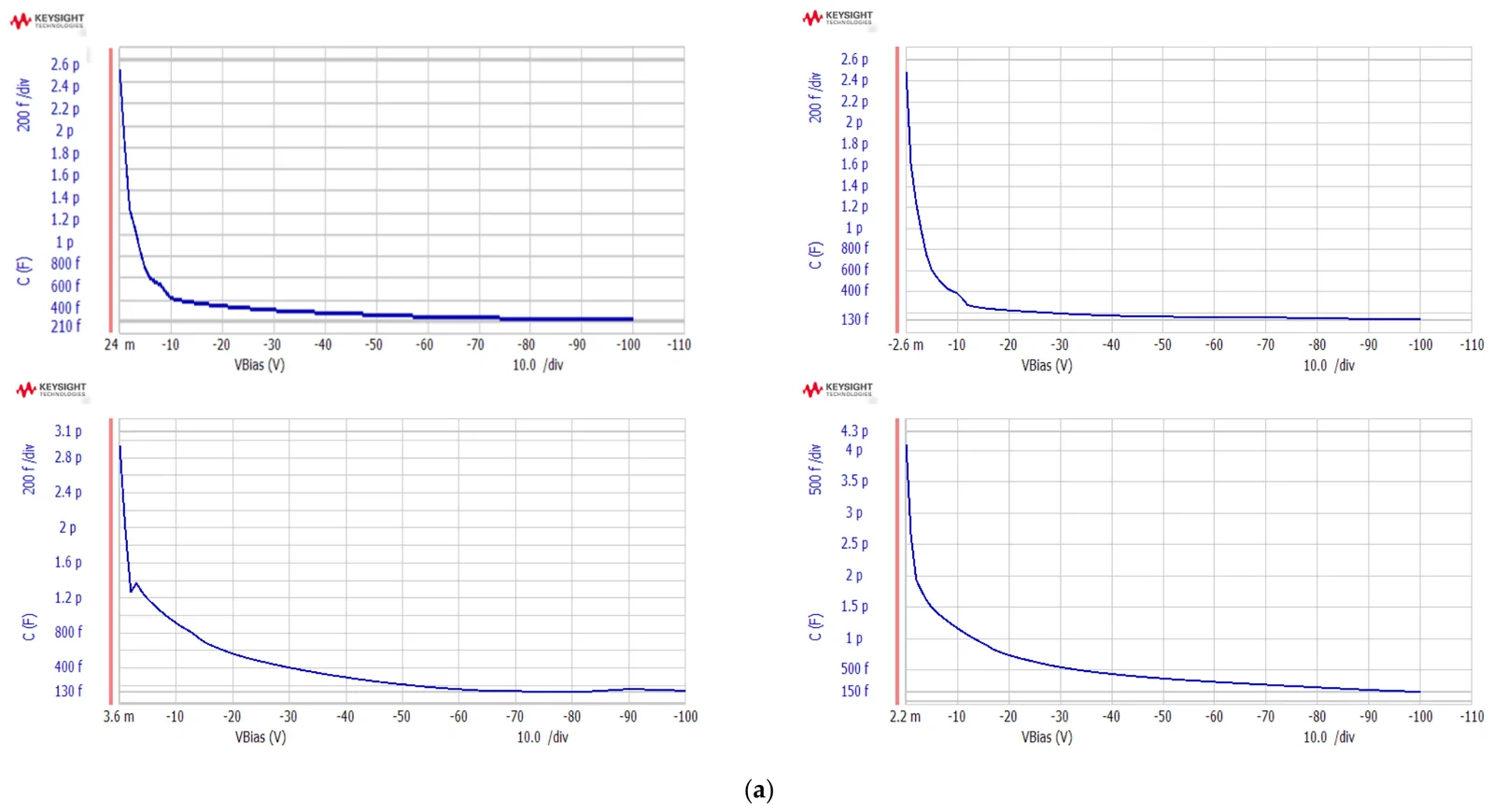

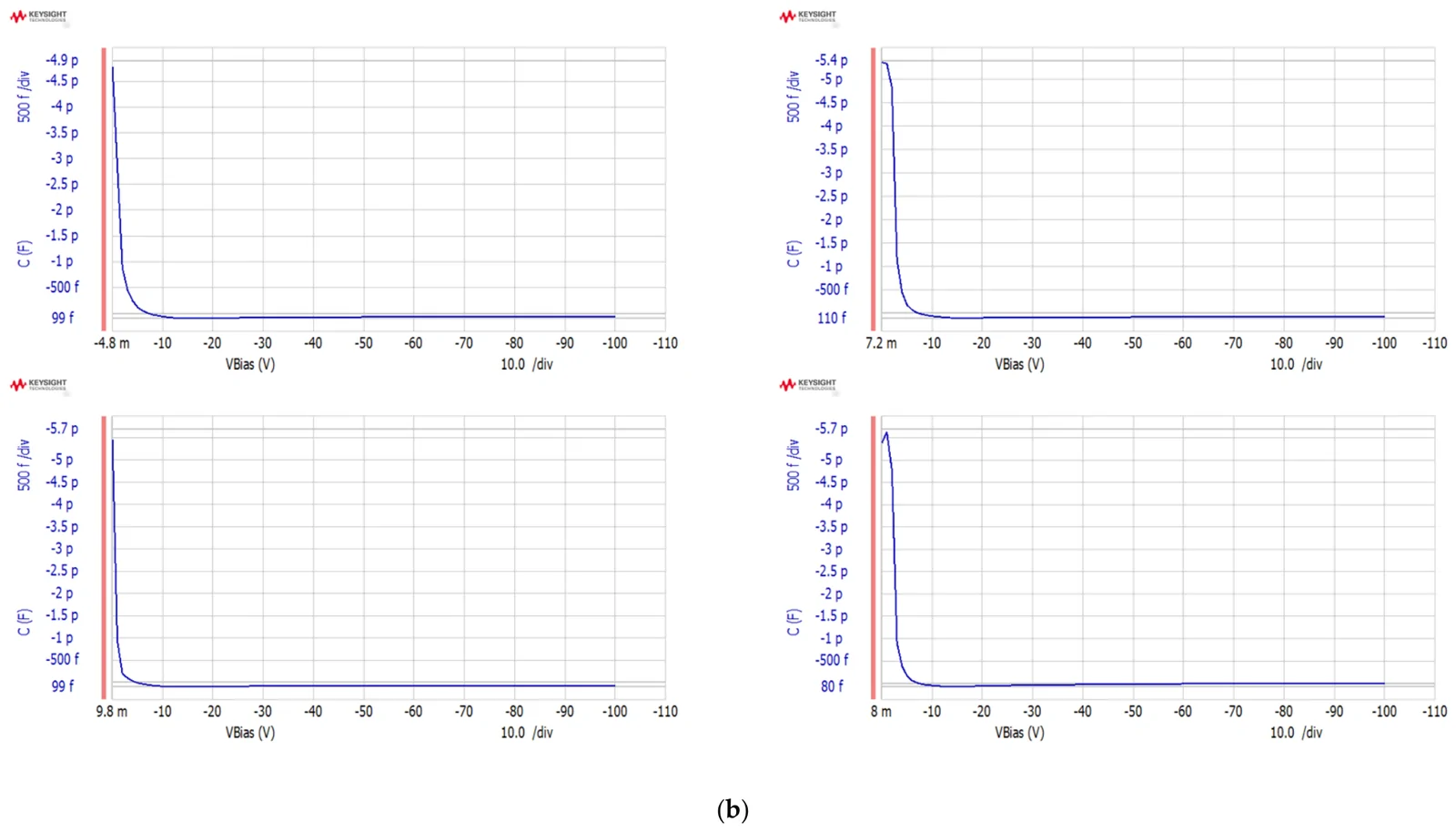
CV Test Curve for (**a**) SDD Sample 1 and (**b**) SDD Sample 2.

All four repeated C-V sweeps for each SDD sample are shown in Figure 15: (a) Sample 1 (anode radius 150 μm) and (b) Sample 2 (anode radius 50 μm). The measured capacitance of Sample 1 is approximately 150 fF, whereas Sample 2 yields about 100 fF, consistent with the expected trend that a smaller anode produces a lower capacitance. Both samples exhibit a clear flat capacitance region beyond the depletion voltage, indicating full depletion.

In the simulation, the extracted capacitance corresponds to the intrinsic geometric capacitance $C_{g}$; in an actual device, the total input capacitance $C_{t}$ also includes parasitic contributions $C_{p}$, i.e., $C_{t}=C_{g}+C_{p}$. These parasitics arise from several sources: (i) the capacitance between the anode readout electrode and surrounding conductors, such as adjacent drift rings, shielding layers, and the substrate; (ii) fringing fields due to finite electrode dimensions; and (iii) charge-related effects at the surface passivation layer (e.g., SiO_2_) and its interface with the silicon bulk.

**Figure 16:**
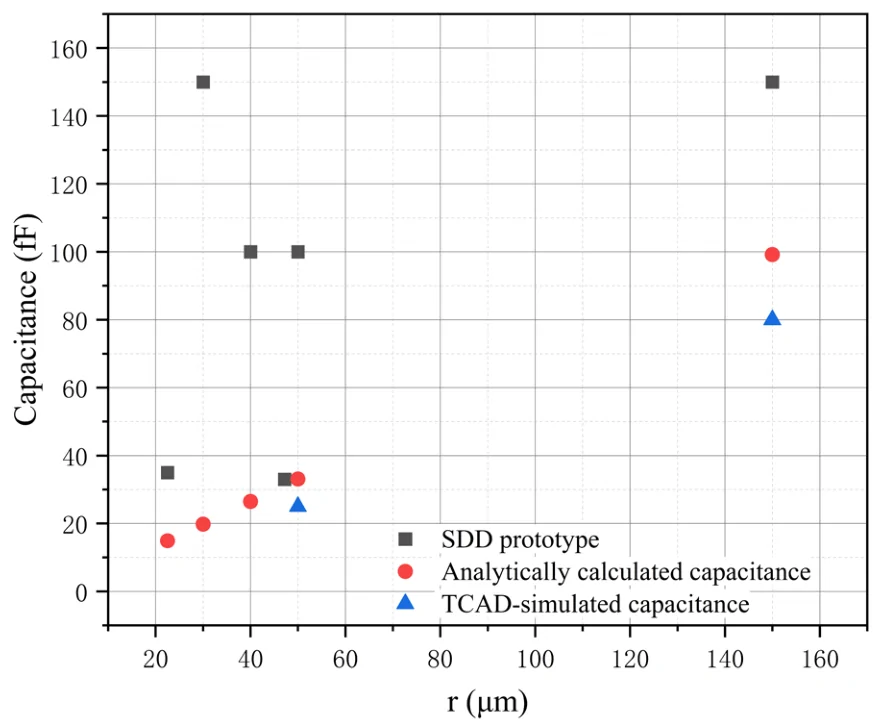
Comparison of calculated, simulated, and measured capacitance for the SDDs.

Figure 16 compares the hemispherical model (Equation (18)), TCAD simulations, and experimental data from our two prototypes together with five mature SDD products from the literature. The geometric parameters and measured capacitances of these devices are summarized in Table 2.

As shown in Figure 16, the TCAD-simulated points closely follow the hemispherical model line $C=2\pi \varepsilon \varepsilon_{0}r_{c}$, with a small systematic offset due to numerical discretization and detailed physical models (e.g., incomplete ionization, fringing fields). Simulations inherently exclude external parasitic contributions. Our two experimental data points and the five literature data points generally fall above the geometric line, consistent with the presence of a parasitic offset. For our two prototypes, the difference between the measured total capacitance and the geometric model prediction is approximately 50–60 fF, which we attribute to the parasitic contribution $C_{p}$. The literature data points, despite originating from different fabrication technologies, detector layouts, and active areas—ranging from 1.8 mm × 1.8 mm to 100 mm^2^—cluster in a similar range of tens of femtofarads above the geometric line when a small anode is employed. This qualitative agreement provides independent support for the anode-dominated scaling law.

We acknowledge that two data points alone cannot rigorously prove a constant parasitic offset, especially given the different outer radii of the two prototypes. The fact that the parasitic estimate is comparable for two devices with substantially different anode radii suggests that $C_{p}$ is not strongly dependent on the anode size. A dedicated test-structure series with multiple anode radii would be required to reliably extract the slope and intercept of the $C_{t}$ − $r_{c}$ relationship.

In practice, minimizing the total capacitance therefore requires both reducing the geometric capacitance, primarily by scaling down the anode radius, and suppressing the aforementioned parasitic components through careful layout design, process optimization, and packaging, so that the overall capacitance approaches the intrinsic limit. A systematic parasitic extraction study using a dedicated test-structure set with multiple anode radii is planned for future work.

## 5. Conclusions

This work has established a unified geometry-based capacitance model for silicon detectors by integrating analytical physical modeling, TCAD simulation, and experimental measurement. The model accurately captures the electrostatic characteristics of planar, 3D trench electrode, 3D spherical electrode, and silicon drift detector (SDD) architectures, and provides explicit analytical expressions linking capacitance directly to the key geometric parameters.

The derived scaling laws reveal fundamental differences among the geometric configurations of these detectors. In planar detectors, capacitance scales with the square of the anode radius and inversely with the depletion thickness. For 3D trench electrode detectors, capacitance increases linearly with electrode depth and depends logarithmically on electrode spacing, enabling a favorable trade-off between low capacitance and low full-depletion voltage. Most strikingly, in highly symmetric structures such as the 3D spherical electrode detector and the SDD, capacitance is dominated almost exclusively by the radius of the collecting anode and is essentially independent of the overall detector volume. This anode-limited behavior, quantitatively described for the SDD by a hemispherical capacitor approximation, represents an ideal low-capacitance limit.

Capacitance measurements on two SDD prototypes with different anode radii confirm the dominant role of the anode size and validate the predictive accuracy of the geometric model. The measured total capacitance can be consistently decomposed into an intrinsic geometric component predicted by the model and a parasitic capacitance arising from interconnects, guard rings, and surface effects. This decomposition, supported by additional data from mature SDD products in the literature, offers a practical pathway for diagnosing and minimizing the total input capacitance in real devices.

By providing a direct cross-structure comparison and explicit capacitance–geometry relationships, this study delivers practical design guidelines for achieving ultra-low input capacitance. Reducing the anode radius is the most effective universal strategy across all investigated structures, while structure-specific adjustments—such as optimizing electrode depth or spacing—offer additional degrees of freedom for the co-optimization of capacitance and operating voltage.

For SDDs, a complete design optimization must also consider the carrier drift time $t_{\mathrm{drift}}\approx R^{2}/(2\mu_{n}V_{\mathrm{drift}})$, which limits the count-rate capability. A hierarchical strategy is recommended: (i) minimize $r_{c}$ to reduce capacitance without affecting drift time; (ii) choose the outer radius R according to the application—small R for high-flux operation, large R for high efficiency; and (iii) maximize the drift voltage within the breakdown limit. For large-area detectors, a multi-anode or pixelated architecture circumvents the $R^{2}$ drift-time bottleneck.

Future work will extend the present model to include temperature-dependent effects, mechanical stress effects, and a systematic parasitic extraction study using dedicated test structures with multiple anode radii. These refinements will enable more accurate performance predictions under realistic and extreme conditions, and will establish comprehensive design guidelines that simultaneously address energy resolution, count-rate capability, and operating voltage, thereby supporting the design of next-generation ultra-low-noise silicon detectors.

## Figures and Tables

**Table 1:** Geometric parameters of the SDD prototypes used in this work.

	Anode Radius r	Thickness d	Unit Radius R
**SDD Sample 1**	150 μm	500 μm	1 cm
**SDD Sample 2**	50 μm	500 μm	0.28 cm

**Table 2:** Geometric parameters and measured capacitance of SDD devices from the literature.

Device	Anode Radius r	Active Area	Capacitance
**Amptek 25 mm^2^ SDD** [27]	22.5 μm	25 mm^2^	35 fF
**Lechner et al. (2001)** [28]	30 μm	100 mm^2^	150 fF
**Fiorini et al. (2006)** [29]	40 μm	10–30 mm^2^	100 fF
**Bertuccio et al. (2024)** [12]	47.2 μm	multiple	33 fF
**Lechner et al. (2001)** [28]	50 μm	5–10 mm^2^	100 fF

## Data Availability

The data presented in this study are available from the corresponding author upon reasonable request.
